# ^1^H-NMR metabolomic study of whole blood from hatchling loggerhead sea turtles (*Caretta caretta*) exposed to crude oil and/or Corexit

**DOI:** 10.1098/rsos.171433

**Published:** 2017-11-22

**Authors:** Stasia A. Bembenek Bailey, Jennifer N. Niemuth, Patricia D. McClellan-Green, Matthew H. Godfrey, Craig A. Harms, Michael K. Stoskopf

**Affiliations:** 1 Department of Clinical Sciences, College of Veterinary Medicine, North Carolina State University, 1060 William Moore Drive, Raleigh, NC 27607, USA; 2 Environmental Medicine Consortium, North Carolina State University, 1060 William Moore Drive, Raleigh, NC 27607, USA; 3 Fisheries, Wildlife, and Conservation Biology, College of Natural Resources, North Carolina State University, 3120 Jordan Hall, Raleigh, NC 27695, USA; 4 Center for Marine Sciences and Technology, North Carolina State University, 303 College Circle, Morehead City, NC 28557, USA; 5 Sea Turtle Project, North Carolina Wildlife Resources Commission, 1507 Ann Street, Beaufort, NC 28516, USA; 6 Nicholas School of the Environment, Duke University Marine Lab, 135 Duke Marine Lab Road, Beaufort, NC 28516, USA

**Keywords:** *Caretta caretta*, Corexit 9500A, crude oil, nuclear magnetic resonance spectroscopy, small volume samples, whole blood

## Abstract

We used proton nuclear magnetic resonance spectroscopy (^1^H-NMR) to evaluate metabolic impacts of environmentally relevant crude oil and Corexit exposures on the physiology of hatchling loggerhead sea turtles (*Caretta caretta*). Sample extraction and data acquisition methods for very small volume whole blood samples and sources of variation between individual hatchlings were assessed. Sixteen unclotted, whole blood samples were obtained from 7-day-old hatchlings after a 4-day cutaneous exposure to either control seawater, crude oil, Corexit 9500A or a combination of crude oil and Corexit 9500A. After extraction, one- and two-dimensional ^1^H-NMR spectra of the samples were obtained, and 17 metabolites were identified and confirmed in the whole blood spectra. Variation among samples due to the concentrations of metabolites 3-hydroxybutyrate, lactate, trimethylamine oxide and propylene glycol did not statistically correlate with treatment group. However, the characterization of the hatchling loggerhead whole blood metabolome provides a foundation for future metabolomic research with sea turtles and a basis for the study of tissues from exposed hatchling sea turtles.

## 1. Introduction

Sea turtles are charismatic species and extremely important to the balance of marine trophic webs [1,2]. However, populations of all seven species are vulnerable to extinction, with several listed as critically endangered by the International Union for Conservation of Nature [3]. Concern has arisen that increasing incidents of oil exposure have the potential to further diminish populations [4–7]. While the direct effects of oil exposure are probably underreported, particularly sublethal and subclinical effects, sea turtles are thought to be sensitive to petroleum exposure at all life stages [8].

Seminal studies in other marine animals have documented sublethal impacts of crude oil on many organ functions, including reproductive effects, adrenal toxicity, cardiotoxicity and lung disease [9–13]. Work with birds demonstrated that the exposure of eggs and newly hatched chicks to very low doses of crude oil has a significant negative impact on the survivability of eggs and newly hatched chicks [14–16]. This is a potential concern for sea turtles. Like nesting shore birds, hatchling sea turtles can be affected by both seasonal fresh spills that contaminate nests and spills that have contaminated nesting areas in the past. However, our understanding of the pathogenesis of conditions attributed to crude oil exposure in young sea turtles remains incomplete [17–22]. Even less is known about the impacts of compounds used to mitigate oil spills on sea turtle metabolism.

In this paper, we describe an evaluation of metabolic impacts of exposure of crude oil and a mixture used in oil spill mitigation, Corexit, on hatchling sea turtles using proton nuclear magnetic resonance (^1^H-NMR) technology. The technique is a highly sensitive tool that can provide insight into the health status through unbiased quantitation of metabolites even in low micro-molar concentrations [23]. ^1^H-NMR techniques provide information distinct from what is obtained through direct measurement of organ-contaminant concentrations or P450-1A activation assays [24–28] and may be better suited to detection of very subtle health effects than routine serum chemistries and haematology [29–31]. A clear understanding of the metabolic impacts of oil exposure on hatchling sea turtles has the potential to improve oil contamination diagnosis, evaluation of treatment protocols and further our understanding of the mechanisms of oil and dispersant toxicity.

To minimize population impacts, we studied eggs from nests unlikely to succeed because of late deposition or their location was vulnerable to destruction. Genomic variability in the study is minimized because all hatchlings in a nest are siblings. Our study was designed to take advantage of the ontogenic pattern of physiologic perturbations caused by petroleum contamination being more extreme in earlier stages of development, a phenomenon documented across a wide taxonomic range of species [32–35]. We assessed the suitability of sample extraction and data acquisition methods for the study of very small whole blood samples and included intraerythrocytic as well as extraerythrocytic metabolites to reduce sample variation due to cellular to extracellular metabolic flux [36]. Sources of variation between individual hatchling metabolomes were also examined.

## 2. Material and methods

### 2.1. Organisms, oil/dispersant exposure and tissue collection

Loggerhead sea turtle eggs were collected from a single nest deemed to be non-viable in the wild due to the high likelihood of complete loss due to timing of lay, nest position, expected weather impacts and predation from invasive species. The choice of using animals from a single nest (all siblings) had the advantage of providing animals with as little genetic variation as feasible, reducing genetic variability in much the way that inbred strains of laboratory animals are valuable in medical research. The eggs were brought to the laboratory and incubated under controlled conditions of 27.2–30.8°C. Prior to hatching, a pseudo-random number generator (Windows Excel, Microsoft Corporation, Redmond, WA, USA) was used to assign the 22 hatchlings to treatment groupings. Each hatchling received its predetermined treatment at 3 days post-hatch, the time expected for nest emergence [37]. The hatchlings were assigned to a 4-day cutaneous exposure in individual glass tubs in one of four exposure groups at concentrations compatible with those observed in Deepwater Horizon oil spill: Crude Oil (Gulf Coast—Mixed Crude Oil Sweet, CAS no. 8002-05-9, 0.833 ml l^−1^ aged seawater), Corexit (Corexit 9500A, 0.083 ml l^−1^ aged seawater), Combined (Gulf Coast—Mixed Crude Oil Sweet, CAS no. 8002-05-9, 0.833 ml l^−1^ and Corexit 9500A, 0.083 ml l^−1^ aged seawater) and Negative Control (exposed only to aged seawater). At the completion of the exposures, animals were euthanized by decapitation and pithing [38]. Whole blood was collected into lithium-heparinized tubes and flash frozen directly in wells of dry ice. The samples were transferred to polyethylene freezer tubes (Thermo Scientific Nalgene, Waltham, MA, USA) and stored at −80°C until further processing.

### 2.2. Metabolite extraction

Although 22 blood samples were made available for our use from the traditional exposure study, samples from six hatchings were not used as the whole blood had clotted in the collection tubes. Metabolomic analysis of the remaining 16 whole blood samples was performed. The sample volume of the 16 unclotted, whole blood samples for this study ranged from 5 to 20 µl (six Negative Control, five Crude Oil, three Corexit and two Combined). To quantitatively assess the whole blood metabolome, including the metabolome of intact erythrocytes, complete haemolysis of samples was achieved by mixing a dilute solution of sodium bicarbonate, potassium ferricyanide and potassium cyanide (Drabkin's Reagent, Ricca Chemical Co., Arlington, TX, USA) with thawed, vortexed whole blood at 10 : 1 (v : v) in a polyethylene tube, using methods validated in our laboratory [39]. After vortexing and a 10 min room temperature (21–22°C) incubation period, the samples were pipetted into Ultracel 10 k centrifugal filters (pre-soaked and rinsed four times with ultrapure water by centrifuge; Merck Millipore Ltd., Carrigtwohill, Co. Cork, Ireland) and centrifuged at 12 848 g for 20 min at 4°C. The filtrates were frozen on dry ice and lyophilized overnight (Labconco FreeZone 2.5 Liter Freeze Dry System, Series 74200, −81.3°C). Following lyophilization, the samples were sealed with Parafilm (Pechiney Plastic Packaging, Chicago, IL, USA) and stored at −80°C for subsequent NMR analysis.

### 2.3. ^1^H-NMR metabolomics and spectral pre-processing

Immediately prior to NMR analysis, the sealed samples were thawed at room temperature and then dissolved in 100% D_2_O containing 0.1 mM trimethylsilyl propanoic acid (TSP), 1 mM formate, 40 mM phosphate buffer and 0.05% sodium azide and filtered through Fisher SureOne filter pipette tips (Fisherbrand SureOne 10 µl, extended, filter, low retention, universal fit pipet tips, Thermo Fisher Scientific, Waltham, MA, USA) at 3000 g for 2 min. One-dimensional ^1^H-NMR spectra of the extracts were obtained using a microcoil NMR probe (Protasis, Marlboro, MA, USA) in a Varian Inova 600 MHz multinuclear INOVA NMR spectrometer (Varian, Inc., Palo Alto, CA, USA) at 25°C with a 1.1 s acquisition time. The sweep width of 7193.6 Hz acquired 8192 complex points and 5120 transients.

### 2.4. Two-dimensional method

To help identify and confirm peaks in the individual hatchling spectra, both one- and two-dimensional experiments were run on combined hatchling whole blood that remained after processing individual samples. The processed samples were dissolved in 300 µl of 10% D_2_O/90% H_2_O containing 0.1 mM TSP, 40 mM phosphate buffer and 0.05% sodium azide and combined before loading in a 3 mm NMR tube (327PP-7; Wilmad-LabGlass, Vineland, NJ, USA). The spectra were obtained using a 5 mm ID ^1^H/BB (^109^Ag-^31^P) Triple-Axis Gradient Probe (ID500-5EB, Nalorac Cryogenic Corp.) in a Bruker 500 MHz NMR spectrometer (Bruker Biospin, Billerica, MA, USA). The one-dimensional experiment (presaturation) was performed with a 2.04 s acquisition time; the sweep width of 8012.82 Hz acquired 16 384 complex points and 256 transients. The two-dimensional experiment, (^1^H–^1^H) total correlation spectroscopy, was performed with (0.13, 0.13) acquisition times; the sweep widths (8005.00, 7996.09) Hz acquired (1024, 2048) complex points and 256 transients.

### 2.5. Spectral pre-processing and quantification

Data were pre-processed using ACD Labs 12.0 1D NMR Processor (Advanced Chemistry Development, Toronto, Ontario, Canada). This included zero-filling to 16 384 points, phase correction, baseline correction and alignment of the reference TSP signal. Although formate was added as an additional standard, TSP was used as the reference on the expectation that protein concentrations were relatively low in these samples [40], and the TSP peaks were consistent and narrow across spectra. For data analysis, residual water and downfield of 9.0 ppm were removed. Two methods of integration were used and compared; intelligent bucket integration with a bin width of 0.03 ppm and integration of identified peaks only. The common integrals for each sample using intelligent bucket integration were normalized by calculating the sum of the integrals for all bins and then dividing each bin by the sum, such that the integrals for each single sample summed to 1. These integrals were subsequently weighted according to sample volume. The common integrals for each sample using identified peaks only were weighted to the TSP integral for each sample and blood volume. Peak identification was performed using Chenomx NMR Suite 8.1 (Chenomx, Edmonton, Alberta, Canada) and The Human Metabolome Database (HMDB; [41]).

### 2.6. Statistical analysis of NMR data

Principal components analysis (PCA) was performed using both intelligent bucket integration and integrals of identified peaks only with JMP Pro12 (SAS Institute, Inc., Cary, NC, USA). For the PCA using intelligent bucket integration, dark regions from −1.5 to 0.5 ppm, 4.7 to 4.9 ppm, and 8.25 to 11 ppm were selected. These dark regions eliminated areas without metabolites, NMR standards and the water peak from analysis. Integrals of identified metabolites were compared among treatments using Kruskal–Wallis tests (Statistix 8.1, Statistix, Inc., Tallahassee, FL).

## 3. Results

### 3.1. Metabolic profiling

**Figure 1.**
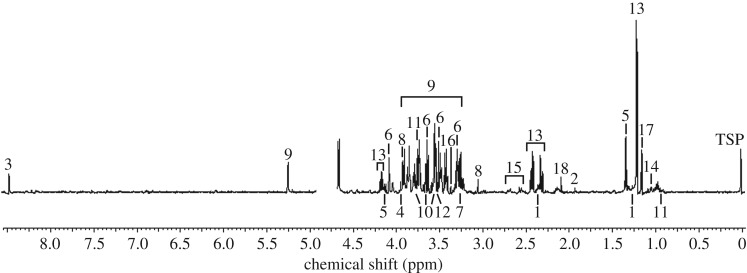
Representative ^1^H-NMR spectrum of an individual hatchling loggerhead sea turtle. The *y*-axis is indicative of peak intensity. The labelled peaks are as follows: (1) 3-hydroxyisovalerate, (2) acetate, (3) formate, (4) glycolate, (5) lactate, (6) myo-inositol, (7) trimethylamine oxide, (8) creatine, (9) glucose, (10) glycerol, (11) alloisoleucine, (12) glycine, (13) 3-hydroxybutyrate, (14) 3-hydroxyisobutyrate, (15) citrate, (16) methanol, (17) propylene glycol, (18) unidentified presumed contaminant.

We identified 17 metabolites from distinct peaks visible in the spectra of hatchling loggerhead sea turtles using one-dimensional ^1^H and two-dimensional ^1^H–^1^H experiments (table 1; figure 1). All samples produced spectra with identifiable peaks. Each metabolite identified occurred in all samples, regardless of treatment group. An unknown contaminant was noted in all samples, including the ‘blank’ samples that contained the rehydration solution only.

**Table 1.** List of metabolites identified in the whole blood of hatchling loggerhead sea turtles using one- and two-dimensional NMR experiments and their respective chemical shifts and multiplicity (d, doublet; dd, doublet of doublets; m, multiplet; s, singlet; t, triplet; tt, triplet of triplets; [41]).

metabolite	^1^H chemical shift and multiplicity
organic acids/osmolytes	
3-hydroxyisovalerate	0.82(d), 0.95(d)
acetate	1.92(s)
formate^a^	8.44(s)
glycolate	3.94(s)
lactate	1.33(d), 4.11(m)
myo-inositol	3.27(t), 3.61(t), 4.05(t), 3.52(dd)
trimethylamine oxide	3.26(s)
energy compounds	
creatine	3.04(s), 3.93(s)
glucose	3.233(dd), 3.398(m), 3.458(m), 3.524(dd), 3.728(m), 3.824(m), 3.889(dd), 4.634(d), 5.223(d)
fatty acid metabolism	
glycerol	3.551(m), 3.644(m), 3.775(tt)
amino acids	
alloisoleucine	0.94(m), 1.38(m), 2.05(m), 3.73(d)
glycine	3.56(s)
ketone bodies	
3-hydroxybutyrate	1.2(d), 2.31(dd), 2.40(dd), 4.15(m)
3-hydroxyisobutyrate	1.114(d), 2.649(m), 3.683(m)
Krebs cycle intermediate	
citrate	2.54(d), 2.68(d)
other	
methanol	3.36(s)
propylene glycol	1.130(d), 3.434 (dd), 3.537(dd), 3.870(m)
unknown^b^	2.06(s)

^a^Greater metabolite concentration noted than expected solely from the reference standard addition. It was also identified in the two-dimensional experiment in which no standard was added.

^b^Unidentified presumed contaminant (seen on rehydration solution ‘blanks’ and in the one-dimensional samples).

### 3.2. Mulivariate statistics

**Figure 2.**
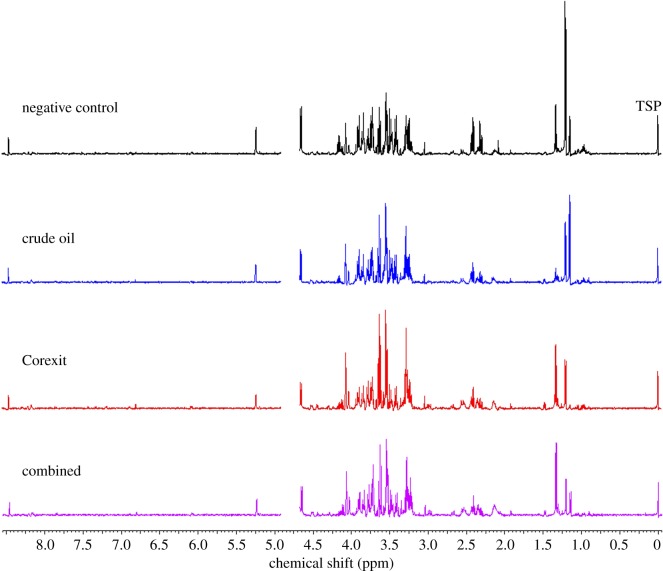
Representative ^1^H-NMR spectra of a hatchling loggerhead sea turtle from each treatment group (negative control, crude oil, Corexit and combined). The *y*-axis is indicative of peak intensity.

The metabolites 3-hydroxybutyrate, lactate, trimethylamine oxide and propylene glycol appeared to be the basis of some variation among the samples, but these differences did not appear to be related to treatment (figure 2). The Kruskal–Wallis tests supported these observations. There were no statistically significant differences between treatment groups (Negative Control, Crude Oil, Corexit and Combined; *p* > 0.05).

**Figure 3.**
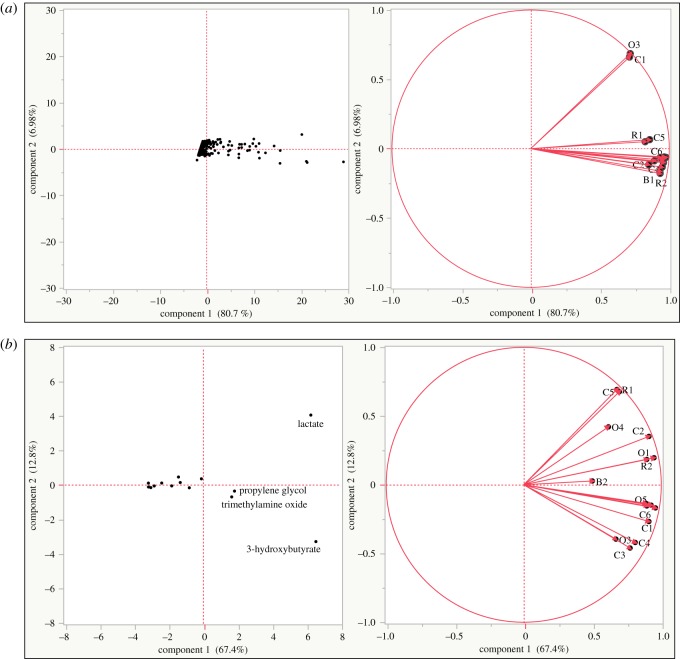
(*a*) PCA summary plots (score plot (left) and loadings plot (right)) of intelligent bins of whole blood spectra of loggerhead sea turtle hatchlings exposed to crude oil (O), Corexit (R), combined (B) and negative control (C). Data were normalized and weighted by blood volume. (*b*) PCA summary plots (score plot (left) and loadings plot (right)) of identified spectral peaks in whole blood of loggerhead sea turtle hatchlings exposed to crude oil (O), Corexit (R), combined (B) and negative control (C). Data were normalized and weighted by blood volume.

When PCA, an unsupervised statistical method often used to illustrate groupings based on similarities in spectral peaks, was applied to the data using intelligent binning (without identification of metabolites), one sample from the control group (15C) and one sample from the oiled group (62O) were found to be more similar to each other than to other samples (figure 3*a*). This unexpected result was not seen when applying PCA using only the data from specific identified metabolites (figure 3*b*).

## 4. Discussion

Although this study did not provide the desired identification of metabolic markers of petroleum exposure in sea turtles, there were important insights into the challenges of NMR metabolomic methods when working with small and valuable samples. The important and unique data from hatchling sea turtles also provided highly useful direction regarding future investigations of biological questions of interest to conservation and sea turtle biologists. Key insights into methodological questions range from information concerning the mathematical treatment of metabolomic spectra, selection of extraction methods and the effects of small sample volume on an environmental metabolomic study.

The mathematical treatment of the raw spectral data, including the choice of statistical approaches for identifying metabolites, can impact the understanding of the results of a metabolomic study. The selection of binning techniques to achieve useful evaluation of sample groupings remains an important issue. Our comparison of use of the commonly used intelligent binning approach with an approach that included only peaks relevant to identified metabolites in PCA analysis illustrates this challenge. Examining the other hatchling data (incubation temperature, date of pip, sex, weight, packed cell volume, total solids) and the individual spectra provided no clear indications why two samples in different treatment groups would group together and separately from the others when intelligent binning was used. This suggests that baseline irregularities or similarity in the low amplitude peaks of insufficient scale for metabolite identification may have been the cause of the unexpected relationship suggested in the intelligent binning PCA.

We had hoped to identify a larger number of metabolites in our samples. Our metabolite yield could be limited by our choice of a relatively non-toxic polar extraction that effectively restricted our ability to assess non-polar metabolites and by extreme constraints in blood volume available for analysis. Blood volume was limited due to the small body size of the subjects (weight range 15.4–18.9 g) as well as the need to preserve blood from the hatchlings for other studies. This constraint may not only have precluded the identification of lower concentration metabolites but also obscured detection of concentration differences in those metabolites. The ability to identify and quantify 17 metabolites in such small samples of hatchling blood does represent an advance in technique. We were able to identify two more metabolites in our small hatchling samples than we were previously able to identify on 50 µl whole blood adult loggerhead samples in which amphibian Ringer's solution was used for extraction rather than Drabkin's solution [42]. This advance is important not only for investigators studying sea turtles, but for others needing information on small species or those where sample access is quite limited. The difference in age class and extraction solvent between the two studies could have contributed to a slightly different profile, and the availability of a two-dimensional spectrum to assist with metabolite identification in this study aided in identifying metabolites. A study using much larger 500 µl plasma samples from juvenile to subadult green sea turtles and a delayed freeze to −80°C and no lyophilization step in the processing reported having identified 55 metabolites [43]. Many of the compounds they reported that we did not see in our spectra were compounds of very low concentration, supporting the expectation that a larger sample size contributes to a greater number of identifiable metabolites. However, our incorporation of a lyophilization step in preparation may also have contributed to loss of low-concentration metabolites in our spectra. One should also consider extraction approaches that are likely to give better yields of non-polar metabolites.

The identified metabolites provide knowledge of metabolism at the early stages of loggerhead sea turtle life and have potentially important implications for understanding sea turtle physiology and development. There were several identified metabolites of particular interest, as they appeared to contribute to variation between individual hatchlings. Among the higher concentration metabolites is 3-hydroxybutrate, which is produced in the liver of many animals. It is a ketone body thought to be used as an energy source in peripheral tissues. In many species, including adult green turtles (*Chelonia mydas*), it accumulates in a fasting state [44–46], but it is possible that it plays a specific role in hatchlings. In newly hatched chickens (*Gallus gallus domesticus*), the blood concentration of 3-hydroxybutrate is five times higher than in 4- and 30-day-old chicks, and the brain actively takes up three times more 3-hydroxybutyrate than 4-day-old chicks [47]. At these early stages of life, ketone bodies, including 3-hydroxybutyrate, are used to a greater extent in the brain than in later stages of life and represent an important energy source and a precursor for amino acids and fatty acids for the brains of young chicks [47]. In mammals, 3-hydroxybutyrate also alters gene expression to increase resistance to oxidative stress [48]. In hatchling sea turtles, it may be that 3-hydroxybutyrate concentrations enhance their ability to manage oxidative stressors such as crude oil and/or Corexit exposure. However, visible differences noted in concentrations of 3-hydroxybutyrate across the hatchling spectra may be related to differing levels of utilization and production by individual animals and not related to treatment.

Lactate also appeared to influence variation between hatchlings in our study based on visual examination of the spectra and PCA. Lactate concentrations in blood increase in many animals, including sea turtles, during high activity [49]. During the critical periods of nest emergence and beach crawling, loggerhead hatchlings have high energy requirements met largely by anaerobic metabolism [50,51]. Blood lactate concentrations of emerging free-ranging hatchlings vary considerably (2.03–12.3 mmol l^−1^, [50]), with most significant differences related to a decrease between beach crawling and 2 h of swimming. However, differences between individuals can be related to time in emergence boil and possibly based on position in the nest cavity and time of hatch related to the emergence from the sand. In our study, although not specifically quantified, there was considerable variation in activity level between hatchlings (C. Harms 2013, personal observation). We expect this natural variation in activity could lead to variable blood lactate concentrations, similar to those observed in the studies performed on wild-caught hatchling sea turtles.

Little is known about the source and time course of accumulation of trimethylamine oxide (TMAO) in marine animals. Although it has been previously identified in hatchling loggerhead turtle blood in our laboratory [51], most of the work on TMAO in marine animals has focused on elasmobranchs and cephalopods. It is a known osmolyte in some species and is thought be a protein stabilizer to counteract the damaging effects of urea in marine animals [52]. Owing to its osmotic properties, it contributes to positive buoyancy in pelagic marine species [53,54]. As such, TMAO could be beneficial for hatchlings through reducing the energy needed for the turtles to maintain their position in the water column.

Previous studies identified propylene glycol in human serum and suggested that it may be an endogenous metabolite, a contaminant or both (e.g. [55,56]). It has further been described as a metabolic by-product of numerous naturally occurring bacteria and yeast. In several species of *Lactobacillus*, propylene glycol was produced through degradation of lactate under anaerobic conditions (reviewed by [57]). In our laboratory, we have found propylene glycol in other sea turtle samples while ruling out sources of sample and processing contamination [58]. This suggests that the propylene glycol is endogenously produced, derived from bacterial production in the gastrointestinal tract of sea turtles, or that it is obtained by the hatchlings from their environment.

We did not find any statistically significant differences in the hatchling metabolomes based on treatment. It may be that the 4-day exposure length was insufficient to result in detectable metabolomic disturbances in our blood samples. However, changes in haematological and plasma chemistry parameters (e.g. leucocytosis, erythrocyte polychromasia and haemoconcentration) have been observed in juvenile loggerhead sea turtles within 4 days of exposure to crude oil [17,32]. Though dermal exposure of oil and/or Corexit has been associated with moderate changes in some haematological parameters, it may be the duration or degree of topical exposure was not sufficient to drive changes in the whole blood metabolome we characterized.

Using age-matched animals from a single, non-viable clutch incubated in a controlled environment after retrieval from the nest allowed us to minimize variation related to environmental factors, including substrate composition, depth of lay, weather prior to egg retrieval and even diet of the laying female. In addition, age, and to the extent possible, genetics and unrelated health factors of the hatchlings were controlled. This choice eliminated the potentially very valuable option of testing multiple doses (duration and concentration) because of the limited number of hatchlings available for study (*n* = 22). As a result, we conclude that the identified metabolites in our study are generally unaffected by cutaneous exposure to crude oil and/or Corexit over 4 days. Small individual differences seen in metabolite concentrations irrespective of treatment status or external influence may be a result of differences in energy storage or usage and individual variation in organ function or cellular metabolism that may change metabolite profiles over minutes-to-hours.

Although we did not find any statistically significant effects of crude oil and Corexit exposure in the blood, it may be that effects on the metabolome will be noted in other hatchling tissues and biofluids. Of particular interest would be the liver with its role in detoxification and biotransformation of xenobiotics [59,60]. Perturbations in metabolites key to biotransformation enzyme function (e.g. glutathione, glycine and acetate) and gluconeogenesis (e.g. pyruvate, alanine and glucose) when hatchlings are exposed to crude oil ± Corexit would be expected. Similarly, the developing heart of many fish species is sensitive to even very low concentrations of crude oil (e.g. [61,62]), suggesting that perturbations in concentrations of metabolites involved in the Cori and tricarboxylic acid cycles (e.g. lactate, pyruvate and succinate) may occur in cardiac tissues after crude oil ± Corexit exposure. Changes in concentrations of metabolites key to maintenance of proper ion channel pore function recently identified as a component of ionotropic and chronotropic impacts of crude oil on cardiac tissue may also become apparent in studies of cardiac tissues [63].

## 5. Conclusion

We conclude that the use of a controlled model of exposure provides benefits in the research of toxicologic effects in hatchling loggerhead sea turtles and that specific useful metabolite profiles can be produced using ^1^H-NMR with very small blood volumes. While differences were not seen between groups in the whole blood metabolome, our findings established a baseline loggerhead hatchling whole blood metabolome that can serve as a foundation for future metabolomic research with sea turtles.

## Data accessibility

Data are maintained by the authors in the data repository of the North Carolina State University Center for Marine Sciences and Technology Marine Magnetic Resonance Facility (https://emc.ncsu.edu/research/facilities/marine-magnetic-resonance/mmrf-data-repository/).
